# Enhanced memory in Wistar rats by virgin coconut oil is associated with increased antioxidative, cholinergic activities and reduced oxidative stress

**DOI:** 10.1080/13880209.2017.1280688

**Published:** 2017-01-24

**Authors:** Nur Syafiqah Rahim, Siong Meng Lim, Vasudevan Mani, Abu Bakar Abdul Majeed, Kalavathy Ramasamy

**Affiliations:** a Collaborative Drug Discovery Research (CDDR) Group, Pharmaceutical and Life Sciences Community of Research, Universiti Teknologi MARA (UiTM), Shah Alam, Selangor Darul Ehsan, Malaysia;; b Faculty of Applied Sciences, Universiti Teknologi MARA (UiTM), Arau, Perlis, Malaysia;; c Faculty of Pharmacy, Universiti Teknologi MARA (UiTM), Selangor Darul Ehsan, Malaysia;; d Department of Pharmacology and Toxicology, College of Pharmacy, Qassim University, Buraidah, Kingdom of Saudi Arabia

**Keywords:** Nutraceutical, neuroprotective, neuroinflammation

## Abstract

**Context:** Virgin coconut oil (VCO) has been reported to possess antioxidative, anti-inflammatory and anti-stress properties.

**Objective:** Capitalizing on these therapeutic effects, this study investigated for the first time the potential of VCO on memory improvement *in vivo*.

**Materials and methods:** Thirty male Wistar rats (7–8 weeks old) were randomly assigned to five groups (*n* = six per group). Treatment groups were administered with 1, 5 and 10 g/kg VCO for 31 days by oral gavages. The cognitive function of treated-rats were assessed using the Morris Water Maze Test. Brains were removed, homogenized and subjected to biochemical analyses of acetylcholine (ACh) and acetylcholinesterase (AChE), antioxidants [superoxide dismutase (SOD), catalase (CAT), glutathione (GSH), glutathione peroxidase (GPx) and glutathione reductase (GRx)], lipid peroxidase [malondialdehyde (MDA)] as well as nitric oxide (NO). α-Tocopherol (αT; 150 mg/kg) was also included for comparison purposes.

**Results:** VCO-fed Wistar rats exhibited significant (*p* < 0.05) improvement of cognitive functions [reduced escape latency (≥ 1.8 s), reduced escape distance (≥ 0.3 m) and increased total time spent on platform (≥ 1 s)]. The findings were accompanied by elevation of ACh (15%), SOD (8%), CAT (≥ 54%), GSH (≥ 20%) and GPx (≥ 12%) and reduction of AChE (≥17%), MDA (> 33%) and NO (≥ 34%). Overall, memory improvement by VCO was comparable to αT.

**Discussion and conclusion:** VCO has the potential to be used as a memory enhancer, the effect of which was mediated, at least in part, through enhanced cholinergic activity, increased antioxidants level and reduced oxidative stress.

## Introduction

Virgin coconut oil (VCO) is increasingly known for its usefulness as functional food oil. This is evident by the increased availability of VCO in the South East Asian countries (Marina et al. 2009b). In general, VCO is predominantly made up of triglycerides (i.e., short- and medium-chain saturated fatty acids). Interestingly, 60% of the medium-chain triglycerides (MCT) in the VCO are almost similar to those in mother’s milk, an ideal natural food formula that protects infants from infections and other illnesses (Isaacs & Thormar 1990). Lauric acid, in particular, is an MCT that encompasses the majority of VCO nutritional content (Bawalan & Chapman 2006). Its amount in VCO is comparable to that in the coconut oil (Marina et al. 2009b). Lauric acid is commonly used as nutritional supplements for infants or patients suffering from malabsorption (Nik Norulaini et al. 2009). Besides, VCO is also rich in active polyphenol compounds, which are strong inhibitors of lipid peroxidation (Dosumu et al. 2010). Polyphenols are known for their neuroprotective actions, especially in preventing the neurotoxic effects of β-amyloid (Menard et al. 2013).

The beneficial effects of VCO have been widely investigated. In fact, VCO has been reported for its excellent antioxidative, anti-inflammatory and antistress properties (Yeap et al. 2015). Capitalizing on these therapeutic effects, the present study assessed the memory-enhancing effect of VCO in normal adult Wistar rats. It is now clear that some aspects of age-related cognitive decline begin in healthy educated adults when they are in their 20s and 30s (Salthouse 2009). Prevention or delay of the progression of age-related cognitive decline would definitely help to reduce the risk of functional impairment. Given that cognitive impairment is correlated with increased vulnerability of various biological components (e.g., lipids, proteins, nucleic acids and cholinergic neurotransmitters) to damaging effects of oxidative stress (Papandreou et al. 2009), VCO appeared to be an ideal nutraceutical-based memory-enhancing agent.

This study also sought to understand the underlying mechanisms of VCO-induced memory-enhancing effects, by which vital insights into the effective use of VCO in the prevention of neurodegeneration would be obtained. The present study focused on the association of VCO-induced memory-enhancing effects with cholinergic activity and oxidative stress process. To the best of our knowledge, this is the first mechanistic study on VCO-induced memory improvement *in vivo*. α-Tocopherol (α-T), a monophenolic lipid-soluble vitamin E, was included in this study for comparison purpose.

## Materials and methods

### Ethics approval

The present animal study was approved by the Committee on Animal Research and Ethics of the Universiti Teknologi MARA (UiTM) [Reference No: 600-FF (PT. 5/2); dated 8 March 2013]. The laboratory animals were handled and managed in accordance to the Guide for the Care and Use of Laboratory Animal (National Research Council 1996).

### Animals

Adult male Wistar rats (7–8 weeks old) weighing between 250–300 g were obtained from the Laboratory Animal Facility and Management (LAFAM) of the Faculty of Pharmacy, UiTM Puncak Alam Campus. The animals were housed under standard laboratory conditions and maintained on a 12 h light/dark cycle with access to food and water *ad libitum*. The rodents were acclimatized to laboratory conditions before the study.

### Treatment groups

**Figure 1.**
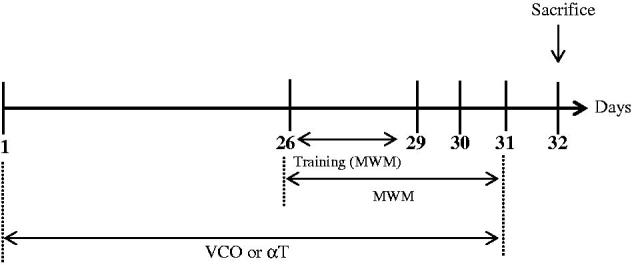
Timeline of treatment and Morris Water Maze (MWM) Test for treatment groups (VCO and αT).

The rats (*n* = 6) were randomly assigned into the following groups: Control (saline only), VCO of 1, 5 and 10 g/kg, respectively (commercially available; Dr Azimuth Formula, Kelantan, Malaysia) as well as α-T [150 mg/kg (Sigma, St Louis, MO)] (Yamada et al. 1999). The VCO, which was prepared via fermentation process, comprised of lauric acid (47.03%), myristic acid (18.71%), caprylic acid (7.93%), palmitic acid (8.86%), capric acid (5.84%), oleic acid (5.52%), stearic acid (3.27%), linoleic acid (0.87%) and caproic acid (1.88%). VCO or αT were administered to the animals *p.o.* for 31 days (Figure 1).

### Morris Water Maze (MWM) Test

The MWM task is a widely accepted method to assess spatial learning and memory of rodents (Morris 1984). Briefly, each rat was exposed to three tracks a day for three days (Day 26–28) before the actual task (Figure 1). For each training trial, each rat was placed at one of the three randomly determined locations and allowed to find the hidden platform. The animal was allowed to remain at the platform for 20 s before being placed in a holding cage for 30 s until the start of the next trial. After training, the actual task was conducted for three consecutive days (Day 29 to Day 31; Figure 1). During the actual task, each rat was placed into the pool at a fixed starting position whereby it was allowed to swim and escape latency, escape distance and swimming speed recorded. The cut off time was 60 s. If the animal crossed the cut off time, it would be guided gently to the platform by hand. Time taken from initial point to platform (escape latency), travelled distance and swimming speed were monitored and analyzed using a video tracking system (ANY-Maze, San Diego Instrument, San Diego, CA). In order to assess memory consolidation, a probe test (day 32) was conducted after the last acquisition session (Figure 1). During this test, platform was removed. Time spent in the platform quadrant was recorded.

### Biochemical assays

Further to behavioural test on day 32, the animals were sacrificed by cervical dislocation (Figure 1). Blood was collected through cardiac puncture. The whole brain was carefully removed from the skull and transferred into phosphate-buffered saline (PBS) before being subjected to homogenization using a glass Wise Stir Homogenizer (Daihan Scientific, Korea). The brain homogenates were centrifuged (2000 *g* at 4 °C for 10 min) and resultant supernatant was collected and stored at −80 °C until biochemical assays were conducted.

#### Acetylcholinesterase (AChE) assay

AChE was measured using the QuantiChrom^TM^ Acetylcholinesterase Assay Kit (BioAssay System, CA) in accordance with the manufacturer’s instruction (Anonymous 2007). Briefly, 10 μL supernatant from brain homogenate of each sample was added into 190 μL working reagent in a 96-well plate. The intensity of the colour change was measured at 412 nm of the 2nd and 10th min using the microplate reader (Tecan, Mannedorf, Switzerland). The activity of this enzyme was determined by calculating the difference of absorbance between the 2nd and the 10th min in comparison to the standard.

#### Acetylcholine (ACh) abundance assay

ACh was measured using the EnzyChrom^TM^ Acethylcholine Assay Kit (BioAssay System, CA) in accordance to the manufacturer’s instruction (Anonymous 2013). Briefly, 20 μL of supernatant from brain tissue lysates was added onto wells containing 80 μL of working reagent. The colour intensity was measured at 570 nm.

#### Superoxide dismutase (SOD) assay

Measurement of SOD activity was performed using the Cayman’s Superoxide Dismutase Assay Kit (Cayman Chemical, Ann Arbor, MI) in accordance with the manufacturer’s instructions (Anonymous 2014e). Briefly, 10 μL of the sample was added into 200 μL radical detector. The reaction was initiated by adding 20 μL xanthine oxidase and the resultant mixture was incubated at room temperature for 20 min on a shaker. The absorbance was measured at 440 nm.

#### Catalase (CAT) assay

Measurement of CAT activity was performed using the Cayman’s Catalase Assay Kit (Cayman Chemical, Ann Arbor, MI) in accordance with the manufacturer’s instructions (Anonymous 2014a). Briefly, 20 μL of the sample was added into 30 μL of methanol and 100 μL of Assay Buffer. The reaction was initiated by adding 20 μL of H_2_O_2_. The mixture was incubated at room temperature for 20 min. To terminate the reaction, 30 μL of potassium hydroxide and purpald chromagen were added and the mixture was incubated at room temperature for 10 min. Finally, 10 μL of potassium periodate was added and incubated at room temperature for 5 min in a shaker. The solution was then measured at 540 nm.

#### Glutathione (GSH) assay

Measurement of GSH concentration was performed using the Cayman’s GSH Assay (Cayman Chemical, Ann Arbor, MI) in accordance with the manufacturer’s instructions (Anonymous 2014b). Briefly, 150 μL freshly prepared assay cocktail containing MES buffer, cofactor mixture, enzyme mixture, water and DTNB was added into 50 μL samples in a 96-well plate. The absorbance was measured at 405 nm and 5 min interval for 30 min.

#### Glutathione peroxidase (GPx) assay

Measurement of GPx was performed using Cayman’s GPx Assay Kit (Cayman Chemical, Ann Arbor, MI) in accordance with the manufacturer’s instructions (Anonymous 2014c). Briefly, 20 μL of the sample was mixed with 100 μL of assay buffer and 50 μL of co-substrate mixture. The reaction was initiated by adding 20 μL cumene hydroperoxide and the absorbance was read every minute (at least 5 time points) at 340 nm.

#### Glutathione reductase (GR) assay

Measurement of GR was performed using the Cayman’s Glutathione Reductase Assay Kit (Cayman Chemical, Ann Arbor, MI) in accordance with the manufacturer’s instructions (Anonymous 2014d). Briefly, 20 μL sample was added into 20 μL GSSG and 100 μL assay buffer. The reactions of the enzyme were initiated by adding 50 μL NADPH to the wells. Absorbance was read at 340 nm every minute (at least 5 time points).

#### Thiobarbituric acid reactive substances (TBARS) assay

The measurement of TBARS, which was essential for screening and monitoring lipid peroxidation, was performed using the Cayman’s TBARS Assay Kit (Cayman Chemical, Ann Arbor, MI) in accordance to the manufacturer’s instructions (Anonymous 2014f). Briefly, 100 μL of the sample was mixed with SDS (sodium dodecyl sulphate) solution and 4 mL colour reagent containing TBA, acetic acid and sodium hydroxide. The solution was then boiled vigorously for 1 h, and immediately cooled down on ice for 10 min to stop the reaction. The solution was centrifuged (2 000 *g* and 4 °C for 10 min). Supernatant was collected and the absorbance was measured at 530 nm.

#### Nitric oxide (NO) assay

Measurement of nitric oxide (NO), a pro-inflammatory mediator, was performed using the QuantiChrom^TM^ Nitric Oxide Assay Kit (BioAssay System, CA) in accordance with the manufacturer’s instructions (Anonymous 2010). Briefly, 100 μL of deproteinated sample was added to 200 μL working reagent containing three mixture of reagent provided by manufacturer. The solution was then incubated at 60 °C for 10 min. After centrifugation (2000 *g* and 4 °C for 10 min), 250 μL supernatant was transferred into 96-well plates and the absorbance was read at 540 nm.

#### Statistical analyses

Statistical significance was analyzed using GraphPad Prism version 6.01 (GraphPad software Inc., La Jolla, CA). Mean differences was evaluated using the One-Way Analysis of Variance (ANOVA) (with *post hoc* Befforoni’s Test). *p* Values <0.05 were considered as statistically significant.

## Results

### VCO-enhanced memory of Wistar rats but not their locomotor

**Figure 2.**
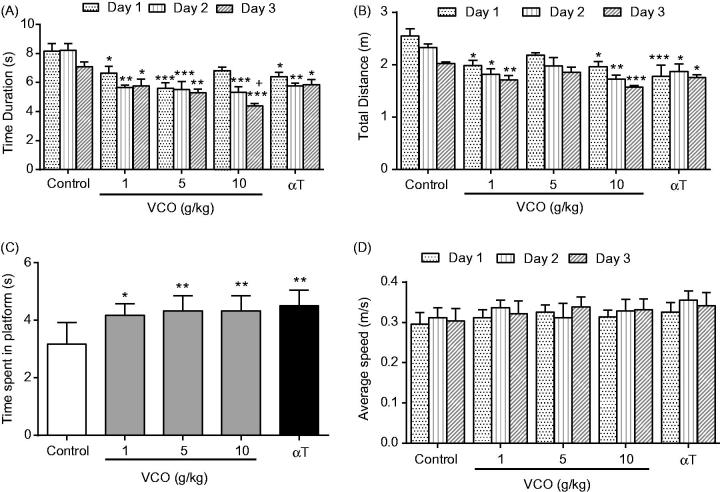
VCO-enhanced memory in Wistar rats. VCO treated rats were subjected to behavioural study using the Morris Water Maze Test. The rodents were assessed as follows: (A) Escape latency, (B) escape distance, (C) time spent by the rat in the escape platform zone (probe test) and (D) average speed taken by the rat to reach escape platform. αT was also included for comparison purposes. Each bar represents mean ± SEM of *n* = 6. *p < 0.05, **p < 0.01, ***p < 0.001 when compared to control group.

Cognitive functions of VCO-treated rats were examined in terms of their escape latency (time to reach platform), escape distance (searching distance) and time spent in platform quadrant (probe test) (Figure 2(A–C)). In addition, the present study also assessed the motor function of treated animals by taking into account the average swimming speed (Figure 2(D)). In general, VCO (1–10 g/kg) significantly (*p* < 0.05) reduced escape latency (Day 1–3) when compared to control (Figure 2(A)). On average, the escape latency of 10 g/kg VCO-treated rats (*p* < 0.001) was reduced by 2.3 s as opposed to that of control (Day 1–3).

The escape distance, on the other hand, was significantly (*p* < 0.05) reduced only in VCO-treated rats at 1 g/kg (by 0.6 m, 0.5 m and 0.3 m; Day 1–3) and 10 g/kg (by 0.6 m, 0.6 m and 0.5 m; Day 1–3) when compared to control (Figure 2(B)). Remarkably, the attenuation of escape latency and escape distance observed among the VCO-treated rodents was comparable to that of αT at day 3 (1.2 s and 0.3 m, respectively). It is noteworthy that 10 g/kg VCO-treated rats exhibited significantly (*p* < 0.05) better escape latency than αT rats at Day 3.

Probe test reflects memory consolidation. VCO (1, 5 and 10 g/kg) and αT (150 mg/kg) were able to significantly (*p* < 0.05) retain the rodents in the platform quadrant for a relatively longer time when compared to control (Figure 2(C)).

In terms of swimming speed, there was no significant difference between the groups. The average swimming speed over three days was ∼0.32 ± 0.01 m/s (Figure 2(D)). This strongly suggest that VCO and αT enhanced only the memory of Wistar rats but not their locomotor activity.

### VCO-improved cholinergic activity in rat brain

**Figure 3.**
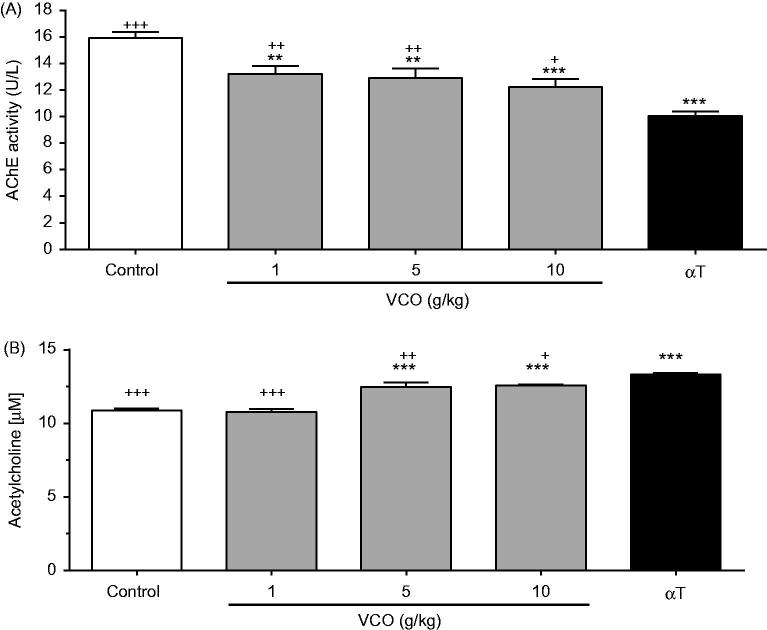
Effects of VCO on cholinergic activity in rat brain. The cholinergic activity in rat brain was assessed: (A) AChE and (B) ACh. Each bar represents mean ± SEM of *n* = 6. *p < 0.05, **p < 0.01, ***p < 0.001 when compared to control; ^+^*p* < 0.05, ^++^*p* < 0.01, ^+++^*p* < 0.001 when compared to αT.

Figure 3(A) illustrates the effects of VCO on AChE activity in rat brain. VCO (1, 5 and 10 g/kg) significantly inhibited AChE activity in a dose-dependent manner when compared to control [17.1% (*p* < 0.01), 19.1% (*p* < 0.01) and 23.2% (*p* < 0.001), respectively]. αT, on the other hand, significantly inhibited AChE by 37.0% (*p* < 0.001) when compared to control. As such, the AChE inhibitory effect of αT was more superior to the highest dose of VCO (10 g/kg). Figure 3(B), on the other hand, shows the effects of VCO on ACh concentration in rat brain. Whilst 1 g/kg showed no effect, 5 and 10 g/kg VCO significantly up-regulated ACh by 14.8% (*p* < 0.001) and 15.4% (*p* < 0.001), respectively, when compared to control. αT, on the other hand, significantly up-regulated ACh by 22.3% (*p* < 0.001) when compared to control. As such, αT was more superior in up-regulating ACh when compared to VCO.

### VCO-enhanced antioxidant activity in rat brain

**Figure 4.**
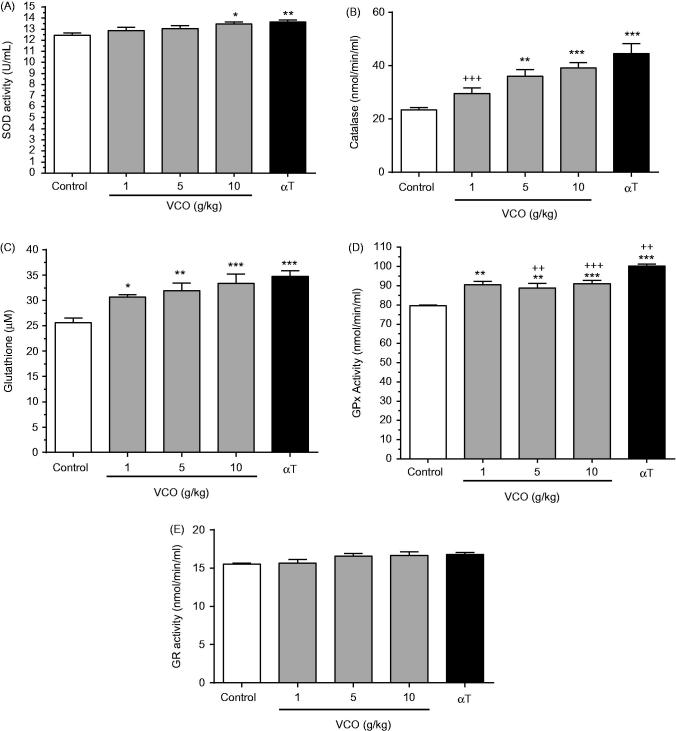
Effects of VCO on antioxidant activity in rat brain. The concentration of antioxidants in rat brain was measured: (A) SOD, (B) CAT, (C) GSH, (D) GPx and (E) GR. Each bar represents mean ± SEM of *n* = 6. *p < 0.05, **p < 0.01, ***p < 0.001 when compared to control; ^+^*p* < 0.05, ^++^*p* < 0.01, ^+++^*p* < 0.001 when compared to αT.

Figure 4 depicts the effects of VCO on antioxidant activity in rat brain. In general, treatment with VCO-enhanced antioxidant level in rat brain. For SOD (Figure 4(A)), while 1 and 5 g/kg VCO showed no effect, 10 g/kg VCO significantly increased its activity by 8.3% (*p < *0.05) when compared to control. αT, on the other hand, enhanced SOD by 9.7% (*p < *0.01) in relative to control. For CAT (Figure 4(B)), whilst 1 g/kg VCO showed no effect, 5 and 10 g/kg VCO significantly up-regulated its activity in a dose-dependent manner [54.0%; (*p* < 0.01) and 67.2% (*p* < 0.001), respectively] when compared to control. αT exerted greater effect, increasing CAT concentration by 90.0% (*p* < 0.001) as opposed to control.

**Figure 5.**
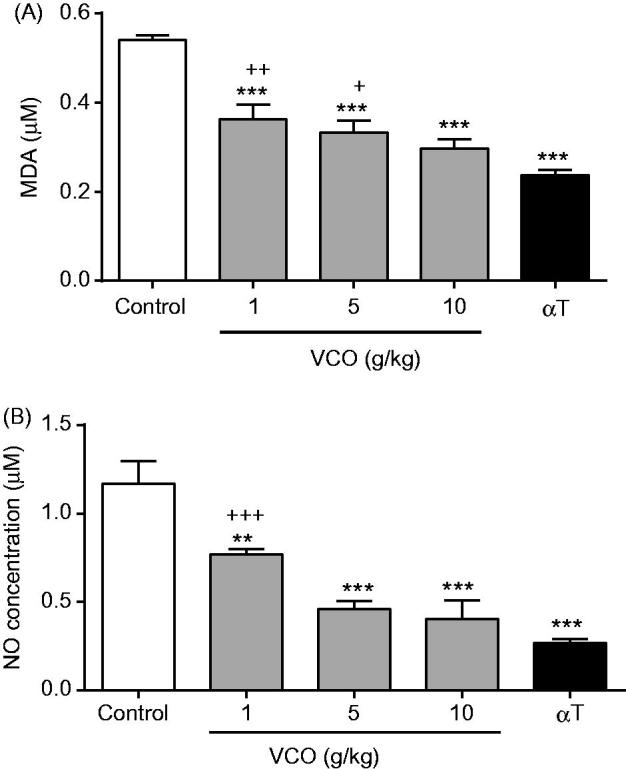
Effects of VCO on oxidative stress in rat brain. The oxidative stress in rat brain was determined: (A) MDA and (B) NO. Each bar represents mean ± SEM of *n* = 6. *p < 0.05, **p < 0.01, ***p < 0.001 when compared to control; ^+^*p* < 0.05, ^++^*p* < 0.01, ^+++^*p* < 0.001 when compared to αT.

For GSH (Figure 4(C)), 1, 5 and 10 g/kg VCO increased its level by 19.7% (*p* < 0.05), 24.8% (*p* < 0.01) and 30.5% (*p* < 0.001), respectively when compared to control. αT enhanced GSH by 35.9% (*p* < 0.001) than control. For GPx (Figure 4(D)), 1, 5 and 10 g/kg VCO increased its level by 13.7%, (*p* < 0.01), 11.7% (*p* < 0.01), 14.3% (*p* < 0.001), respectively, when compared to control. αT significantly increased GPx activity by 26.1% (*p* < 0.001; almost twofold of VCO) in rat brain as opposed to control. For GR (Figure 4(E)), there was no significant difference between treatments.

Overall, VCO-enhanced antioxidant activity in rat brain was almost comparable to that of αT. Significant differences between VCO and αT were only observed in catalase [1 g/kg VCO only] and GPx [1–5 g/kg VCO only].

### VCO-reduced oxidative stress level in rat brain

Figure 5 highlights the effect of VCO on oxidative stress level in rat brain. In general, treatment with VCO-reduced oxidative level in rats. For MDA (Figure 5(A)), 1, 5 and 10 g/kg VCO significantly (*p* < 0.001) inhibited its level in a dose-dependent manner (by 32.8, 38.3 and 45.1%, respectively) when compared to control. αT, on the other hand, significantly inhibited MDA level by 56.3% (*p* < 0.001) as opposed to control. Significant differences were observed between the lower doses of VCO (1 and 5 g/kg) and αT but not at 10 g/kg VCO. For NO (Figure 5(B)), 1, 5 and 10 g/kg VCO significantly inhibited its level in a dose-dependent manner [by 34.4% (*p* < 0.01), 60.6% (*p* < 0.001) and 65.4% (*p* < 0.001), respectively] when compared to control. αT, on the other hand, significantly inhibited NO level by 77.1% (*p* < 0.001). No significant differences were observed between VCO (5 and 10 g/kg) and αT.

## Discussion

The present study investigated the potential use of VCO as a natural memory enhancer in normal adult Wistar rats. The MWM was first performed to evaluate the spatial memory function of VCO-treated rats. The behavioural test requires subjects to search for a hidden platform in opaque water and memorize the location of the platform (Li et al. 2011). The present study demonstrated for the first time that treatment with VCO enhanced and consolidated memory of tested rodents. The VCO-induced memory-enhancing effect could be due to the presence of ketones. VCO, a natural source of medium-chain triglyceride (MCT), is readily oxidized to ketones. Ketone is known to supply a rapid alternative source of energy to the neurons (Sabitha & Vasudeva 2010; Gandotra et al. 2014). It is a short-chain fatty acid that can readily cross the blood–brain barrier to be absorbed by neuron and oxidized in the mitochondria to generate ATP (energy) (Page et al. 2009; Fernando et al. 2015). Interestingly, the memory-enhancing effect of VCO was comparable to that of αT. αT is a monophenolic lipid-soluble Vitamin E that protects cell membranes against lipid peroxidation (Bharrhan et al. 2010). Its *in vivo* memory-enhancing effect appeared to vary among reports. Morris et al. (2015) reported that αT acetate significantly improved learning in normal adult Wistar rat by reducing escape latency and exhibiting higher target quadrant preference in the probe test. Shichiri et al. (2011), on the contrary, found αT to show no significant cognitive improvement in normal adult Wistar rat model. Nevertheless, in a recent clinical study, αT was reported to slow down disease progression in AD patients. Sano et al. (1997) found that treatment with αT (2000 IU a day) reduced neuronal damage and slowed disease progression of patients with moderately severe impairment from AD.

The memory-enhancing effect of VCO was found to be accompanied by increased cholinergic activity in rat brain. The present study found that VCO inhibited AChE activity and at the same time enhanced ACh in treated rats. ACh is a cholinergic neurotransmitter that plays important role in regulating the cognitive functions of brain. Increased AChE activity, however, hydrolyzes ACh into acetate and choline, resulting in the termination of synaptic transmission (Zugno et al. 2014). Anwar et al. (2012) reported that phenolic compounds decreased AChE activity in the cerebral cortex and striatum of adult Wistar rats. As such, the improved cholinergic activity in this study might also be attributed to phenolic compounds in VCO (Marina et al. 2009a). The VCO-enhanced cholinergic activity was also consistent with that of αT. Nevertheless, the effect of αT on cholinergic activity could be dose-dependent. Previous report found that lower concentration of 50 mg/kg αT did not alter AChE activity in normal Wistar rat brain (Gutierres et al. 2012).

VCO-derived biologically active compounds such as polyphenols, tocopherols, sterols and squalene are known for their potent antioxidative activity (Abujazia et al. 2012). In parallel to this, the present findings indicated increased antioxidants and decreased oxidative stress in rat brains. This strongly suggested that VCO-enhanced memory was closely associated with changes of antioxidant status in brain. Oxidative stress is postulated to be a major causal factor of senescence. Accumulation of oxidative stress such as ROS in tissues during aging process modifies macromolecules including protein, DNA and lipid (Sasaki 2010). Antioxidants like SOD, CAT, GSH, GPx and GR are mutually supportive team of defence against oxidative assault by ROS and lipid peroxidation (Priya et al. 2011; Adhikari & Arora 2014). Catechin is a polyphenolic compound that is found in VCO (Marina et al. 2009a). It has been reported to elevate the activity of SOD and CAT in mice striatum (Mandel et al. 2008). Elsewhere, polyphenol fraction extracted from VCO enhanced antioxidant status and reduced lipid peroxide content in hearts, livers and kidneys of Sprague-Dawley rats (Nevin & Rajamohan 2006). In addition, it was reported that the presence of cerebral ketones was associated with reduction of apoptosis, inflammation and oxidative stress (Krikorian et al. 2012). The antioxidative capacity of ketone (Veech et al. 2001; Ziegler et al. 2003; Krikorian et al. 2012) may have also contributed to the attentuation of ROS by VCO as observed in this study. Similar antioxidative effect was also observed amongst αT-treated rats. Previous study found αT to reduce hepatic MDA level and restored CAT and GPx activity in aged Wistar rat (Helmy 2012). αT is able to detoxify H_2_O_2_ by converting it to O_2_ and H_2_O under physiological conditions (Feng & Wang 2012). Inhibition of free radicals formation generated from lipid peroxidation by αT prevented complete oxidation (Dumont & Beal 2011).

## Conclusions

**Figure 6.**
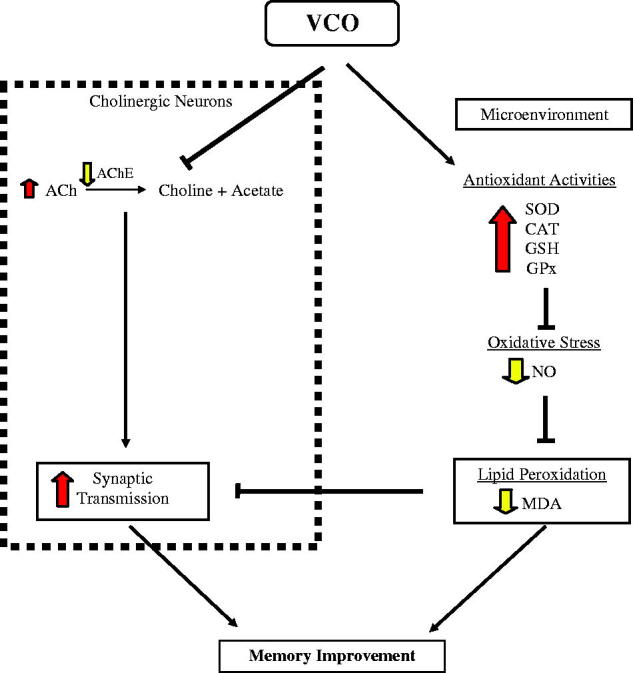
Proposed mechanisms underlying VCO-induced memory improvement in normal adult Wistar rats. The enhanced memory under the influence of VCO is likely mediated through two biological components in the brain: cholinergic neurons and the microenvironment. For cholinergic neurons, VCO reduced AChE action which in turn increased ACh concentration in the brain. The increased ACh plays important role in effective synaptic transmission during acquiring new information and consolidation of memory. Within the microenvironment, on the other hand, VCO increased production of antioxidants in brain, namely SOD, CAT, GSH and GPx. The up-regulated antioxidants served as protection against lipid peroxidation (MDA) by oxidative stress (NO). The collective effects of these two components would eventually prevent or delay neurodegeneration.

Altogether, findings from the present animal behavioural study indicated that both VCO and αT enhanced cognitive function of normal adult Wistar rats. Nevertheless, the observed effect was not accompanied by apparent changes in locomotor function. The memory-enhancing effect of VCO appeared to be more apparent at 10 g/kg and above. The enhanced memory under the influence of VCO is likely mediated through two biological components in the brain (Figure 6): cholinergic neurons and the microenvironment. For cholinergic neurons, VCO reduced AChE action which in turn increased ACh concentration in the brain. The increased ACh plays important role in effective synaptic transmission during acquiring new information and consolidation of memory. Within the microenvironment, on the other hand, VCO increased production of antioxidants in brain, namely SOD, CAT, GSH and GPx. The up-regulated antioxidants served as protection against lipid peroxidation (MDA) by oxidative stress (NO). The collective effects of these two components would eventually prevent or delay neurodegeneration. The promising outcomes of this study strongly imply the possible use of VCO, not only as neuroprotective agents for those suffering from neurodegenerative diseases, but also as brain food (supplements for the health populations).
